# Surface Oxygen Species in Metal Oxide Photoanodes for Solar Energy Conversion

**DOI:** 10.3390/nano13131919

**Published:** 2023-06-23

**Authors:** Jie Ouyang, Qi-Chao Lu, Sheng Shen, Shuang-Feng Yin

**Affiliations:** State Key Laboratory of Chemo/Biosensing and Chemometrics, Advanced Catalytic Engineering Research Center of the Ministry of Education, College of Chemistry and Chemical Engineering, Hunan University, Changsha 410082, China

**Keywords:** photoanode, metal oxide, surface oxygen species, solar energy conversion

## Abstract

Converting and storing solar energy directly as chemical energy through photoelectrochemical devices are promising strategies to replace fossil fuels. Metal oxides are commonly used as photoanode materials, but they still encounter challenges such as limited light absorption, inefficient charge separation, sluggish surface reactions, and insufficient stability. The regulation of surface oxygen species on metal oxide photoanodes has emerged as a critical strategy to modulate molecular and charge dynamics at the reaction interface. However, the precise role of surface oxygen species in metal oxide photoanodes remains ambiguous. The review focuses on elucidating the formation and regulation mechanisms of various surface oxygen species in metal oxides, their advantages and disadvantages in photoelectrochemical reactions, and the characterization methods employed to investigate them. Additionally, the article discusses emerging opportunities and potential hurdles in the regulation of surface oxygen species. By shedding light on the significance of surface oxygen species, this review aims to advance our understanding of their impact on metal oxide photoanodes, paving the way for the design of more efficient and stable photoelectrochemical devices.

## 1. Introduction

The increasing demand for sustainable and clean energy has led to an intense focus on the development of efficient solar energy conversion systems [1]. The use of photoelectrochemical devices, such as photoelectrochemical cells (PECs), for directly storing intermittent solar energy as chemical energy, is a promising solution [2]. PECs can produce chemical fuels (such as hydrogen and methane) from abundant raw materials such as water and carbon dioxide under sunlight, thus reducing our dependence on fossil fuels and having far-reaching effects on slowing down climate change and enhancing energy security [3,4]. In recent years, research and development in this field have aimed to improve the feasibility and practicality of these devices [5]. The most important part is the semiconductor photoelectrode that combines the functions of light absorption, charge separation, and surface chemical reaction [6].

In PECs, the electron–hole pairs produced by the photoelectrode undergo reduction and oxidation reactions at the cathode and anode, respectively. In the actual photoelectrochemical production of sustainable fuels and chemicals, whether it is hydrogen production from water or the reduction of carbon dioxide or nitrogen, consuming electrons and protons at the photocathode needs to be matched with the photoanode to drive water oxidation or other substrates’ oxidation [7]. This necessitates the development of efficient, stable, and cost-effective PEC photoanodes. The successful assembly of PEC devices with high solar-chemical conversion efficiency greatly depends on the advancement of such photoanodes.

**Figure 1:**
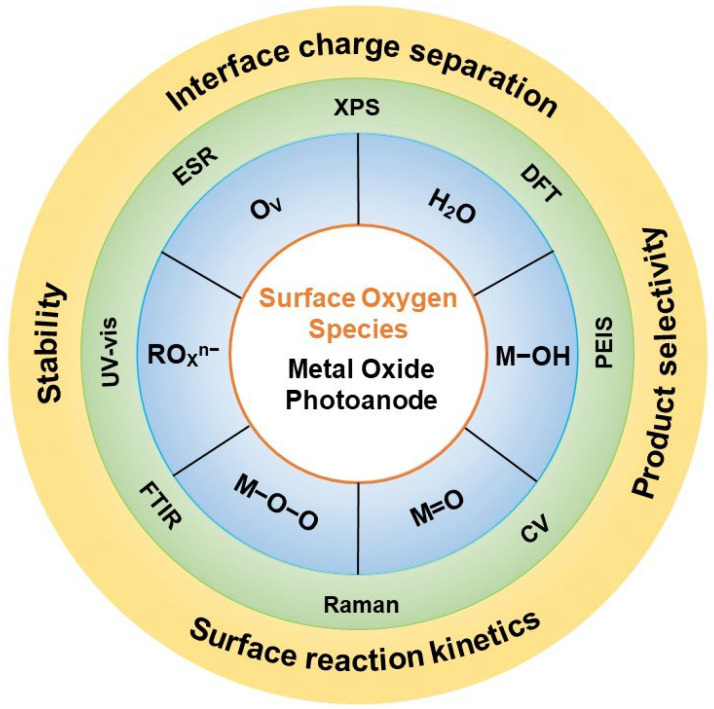
Overview of points discussed in this review.

Metal oxides, such as BiVO_4_, TiO_2_, Fe_2_O_3_, and WO_3_, have emerged as promising materials for photoanodes due to their unique electronic structure properties, good stability, and abundance [8]. Despite these advantageous qualities, these materials suffer from drawbacks including low absorption in the visible-light range, poor charge transfer, and slow surface reactions, resulting in low solar energy conversion efficiency [9]. In recent years, various strategies have been employed to enhance the performance of metal oxide photoanodes, including morphology control [10], crystal facet engineering [11], ion doping [12,13], defect engineering [14], and core–shell structure construction [15]. However, it should be noted that most of these approaches impact the state of surface oxygen species in the photoanode, which plays a critical role in charge separation and surface reactions. Despite notable advancements in the field of surface oxygen species on photoanodes, a comprehensive literature review addressing this topic is currently lacking. Therefore, the primary objective of this article is to fill this gap by presenting a comprehensive and up-to-date overview of surface oxygen species on metal oxide photoanodes (Figure 1). Through a critical assessment of existing research, this review aims to identify the primary challenges and potential pathways for future exploration in the realm of surface oxygen species, with the ultimate goal of advancing sustainable energy production.

## 2. Surface Oxygen Species

To enhance the efficiency of photoelectrochemical systems, a thorough understanding and precise control of the surface properties of metal oxide photoanodes are crucial. Among the various factors influencing the photoelectrocatalytic performance of these photoanodes, surface oxygen species play a particularly significant role. These surface oxygen species can profoundly impact the reactivity, stability, and photoelectrochemical properties of metal oxide photoanodes. Specifically, surface oxygen vacancies, which are oxygen-related surface structures that influence the surrounding metal coordination environment, have been extensively studied as crucial reaction sites. Therefore, in this article, we acknowledge surface oxygen vacancies as a distinct and noteworthy type of surface oxygen species.

Additionally, we focus on exploring oxygenated species present on the surface of metal oxides as another important category of surface oxygen species. In this section, we provide a detailed overview of surface oxygen vacancies and oxygenated species in metal oxide photoanodes. Specifically, we discuss the classification of these surface oxygen species and delve into their respective generation mechanisms. Furthermore, we examine the advantages and disadvantages associated with their presence in photoelectrocatalytic processes.

### 2.1. Surface Oxygen Vacancies

Vacancies are inherent in metal oxides and can be categorized into metal vacancies and oxygen vacancies based on the type of missing ions. Surface oxygen vacancies specifically pertain to defects occurring within the lattice of oxygen atoms on the surface of metal oxides. The formation of surface oxygen vacancies primarily stems from the behavior of oxygen atoms within the solid structure. Two mechanisms commonly contribute to the generation of vacancies on the solid surface: 1. Differences in lattice structure, morphology, or composition of the material surface from the bulk can lead to a scarcity of oxygen atoms on the surface [16]. 2. The interaction between the material surface and the surrounding environment can cause some surface oxygen atoms to detach from the material’s surface [17]. These mechanisms collectively contribute to the presence of surface oxygen vacancies in metal oxide photoanodes.

The introduction of surface oxygen vacancies induces changes in the electronic structure and band gap of metal oxides. By regulating surface oxygen vacancies, it is possible to adjust charge transport at the interface and increase the participation of holes in surface reactions [18]. Moreover, it allows for the modulation of adsorption of the reaction substrate, altering the energy of reaction intermediates and thereby influencing surface reaction kinetics and product selectivity [19].

However, it is important to consider the disadvantages associated with surface oxygen vacancies. A high concentration of oxygen vacancies can create intermediate energy gap states within the photoanode, resulting in a more pronounced recombination and reduced photocatalytic activity [18]. Furthermore, in some cases, surface oxygen vacancies can destabilize the oxide structure, leading to degradation.

To enhance the efficiency of the photoanode’s reaction, it is imperative to gain a clearer understanding of the role of surface oxygen vacancies in photoelectrochemistry. By carefully analyzing the structure–activity relationship between surface vacancies and activity, we can provide refined guidelines and design strategies for the regulation of oxygen vacancies. In this section, we primarily discuss the construction strategies for surface oxygen vacancies in metal oxides and their role in photoelectrocatalysis.

#### 2.1.1. Modulation Strategies

##### Heat Treatment

Heat treatment is a commonly employed method to introduce oxygen defects in metal oxides. It involves subjecting metal oxides to elevated temperatures in a reducing gas atmosphere, where reducing gases react with the metal oxides, resulting in the removal of some lattice oxygen atoms and the introduction of oxygen defects. H_2_ is often used as the reducing atmosphere. For example, Li achieved non-stoichiometric WO_3-X_ by calcining a tungsten oxide photoanode in an H_2_/Ar atmosphere [20]. The content of oxygen vacancies could be controlled by adjusting the calcination temperature. The resulting WO_3-X_ photoanode, with an optimal amount of oxygen vacancies and calcined at 350 °C, exhibited a ten-fold increase in photocurrent density compared to WO_3_, along with excellent catalytic stability. It has been observed that non-stoichiometric WO_3-x_ obtained through hydrogen treatment is resistant to reoxidation and can prevent the dissolution of peroxy species induced by oxidation.

**Figure 2:**
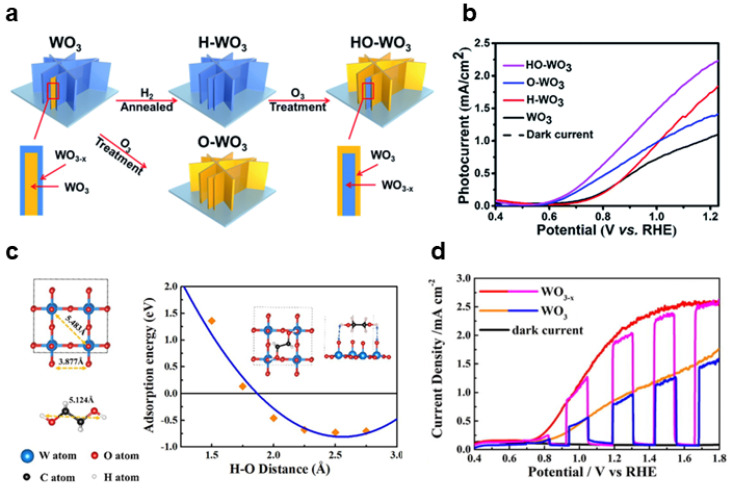
(**a**) Schematic of the structure and synthesis process of WO_3_, H–WO_3_, O–WO_3_, and HO–WO_3_; (**b**) J–V curves of WO_3_, H–WO_3_, O–WO_3_, and HO–WO_3_ in 0.1 M Na_2_SO_4_ under 100 mW cm^−2^ AM 1.5G. Reproduced with permission [21]. Copyright: 2018, Royal Society of Chemistry. (**c**) Top view of the WO_3_ surface; structure of an ethylene glycol molecule; energy, top view, and side view of an ethylene glycol molecule adsorbed by the WO_3_ surface; (**d**) J–V curves of WO_3_ and WO_3−x_. Reproduced with permission [28]. Copyright: 2021, Elsevier.

The introduction of surface vacancies in metal oxides often results in the simultaneous introduction of bulk vacancies. However, effectively controlling the content and type of vacancies in metal oxides presents certain challenges, as different vacancies can have diverse or even contradictory effects on the photoelectrochemical properties. Moreover, an excess of vacancies could potentially hinder catalytic activity, emphasizing the need for precise regulation in order to achieve optimal performance. For example, Gong et al. showed that hydrogen-treated WO_3_ has a large number of oxygen vacancies both on the surface and in the bulk, but only the surface oxygen vacancies acted as charge recombination centers [21]. They demonstrated through experimentation that ozone treatment could remove the surface oxygen vacancies and enhance the catalytic activity of WO_3_ (Figure 2a, b). Similarly, research by Li and Chen indicated that the existence of surface oxygen vacancies in WO_3_ was related to charge recombination [22]. As the number of surface oxygen vacancies increased, the interface charge transfer rate constant and charge recombination rate constant also increased on the WO_3_ photoanode surface. In addition, as the number of oxygen vacancies in the WO_3_ bulk increased, the electrode conductivity and density of trapping sites also increased, affecting the charge separation and transfer. Therefore, the number of oxygen vacancies in the WO_3_ bulk and surface has an optimal value for maximizing the charge transfer and injection efficiency. By using a two-step flame heating process, they can adjust both bulk and surface phase oxygen vacancies, resulting in a tenfold increase in the water oxidation photocurrent density of the optimized WO_3_ photoanode. The strategy of introducing oxygen vacancies into metal oxides by calcining in a reducing atmosphere is not only commonly used for modifying WO_3_, but also studied in TiO_2_ [23], Fe_2_O_3_ [24,25], and BiVO_4_ [26,27] photoanodes. Depending on the differences in materials, suitable amounts of oxygen vacancies need to be introduced into the material under different temperatures and gas flows during calcination.

While reducing atmosphere calcination typically requires high temperatures and enables the introduction of both oxygen vacancies and bulk defects in a single step, there is considerable interest in exploring simple and convenient methods at lower temperatures to control the introduction of either bulk or surface defects in metal oxides.

The solvothermal method is a commonly employed technique for preparing surface oxygen defects, offering lower treatment temperatures and a greater abundance of surface oxygen defects. For example, Wang et al. treated a WO_3_ photoanode with an ethylene glycol solution at 130 °C for solvothermal treatment, leading to the introduction of an appropriate number of oxygen vacancies [28]. Density functional theory calculations showed that the distance between the two hydrogen atoms of the alcohol group in ethylene glycol (5.124 Å) matches the distance between the oxygen anions on the WO_3_(002) surface (5.483 Å). This allowed ethylene glycol to form stable bifurcated hydrogen bonds with O-H groups, with a distance of approximately 2.5 Å (Figure 2c). Due to the unique hydrogen bond structure of ethylene glycol and the moderate reduction environment under heating conditions, an appropriate number of surface oxygen vacancies can be introduced into WO_3_, and its water oxidation activity is improved (Figure 2d). Similarly, Fe_2_O_3_ can also be treated with ethylene glycol solvent heat treatment to introduce rich surface oxygen vacancies [29].

##### Chemical Reduction

To induce oxygen defects in metal oxides at room temperature, researchers have explored the use of reductive reagents. By choosing reagents with high reductive properties, oxygen vacancies can be introduced on the surface of metal oxides without the need for high temperature. Park et al. employed lithium dissolved in ethylenediamine (LEDA) to treat a WO_3_ photoanode for a short duration, resulting in the formation of WO_3_ with both oxygen and tungsten vacancies, which leads to an increase in the density of free carriers [30]. This enhances interfacial charge transmission and improves conductivity. Consequently, the activity of the WO_3_ photoanode is significantly improved.

Another commonly used reduction reagent is NaBH_4_ solution. When a BiVO_4_ photoanode is immersed in NaBH_4_ solution, the Bi-O bonds on its surface break, leading to the removal of oxygen atoms and the formation of oxygen vacancies [31]. Furthermore, treatment with hypophosphorous acid has been employed to introduce a suitable number of oxygen vacancies in BiVO_4_ photoanodes, optimizing the performance of the photoanode [32]. These alternative reduction methods provide a way to construct oxygen defects in metal oxides at room temperature. By utilizing reductive reagents such as LEDA, NaBH_4_ solution, or hypophosphorous acid, researchers can achieve the desired defect structures in metal oxide photoanodes, leading to enhanced charge transfer, improved conductivity, and better catalytic performance.

##### Photo/Electrochemical Treatment

In addition to the methods mentioned earlier, electrochemical reduction offers a milder and more environmentally friendly approach to generate oxygen vacancies in metal oxide photoanodes [33]. For example, Wang et al. utilized electrochemical reduction by applying a potential of − 0.1 V_RHE_ in a potassium borate solution to reduce BiVO_4_ for 150 s [33]. This process led to the partial reduction of Bi^3+^ and V^5+^ ions in BiVO_4_, resulting in the generation of oxygen vacancies. They found that the introduction of oxygen vacancies enhanced the separation efficiency between the bulk phase and the surface of BiVO_4_, thereby improving its water oxidation performance. This electrochemical reduction method was also applied to TiO_2_ photoanodes [34].

**Figure 3:**
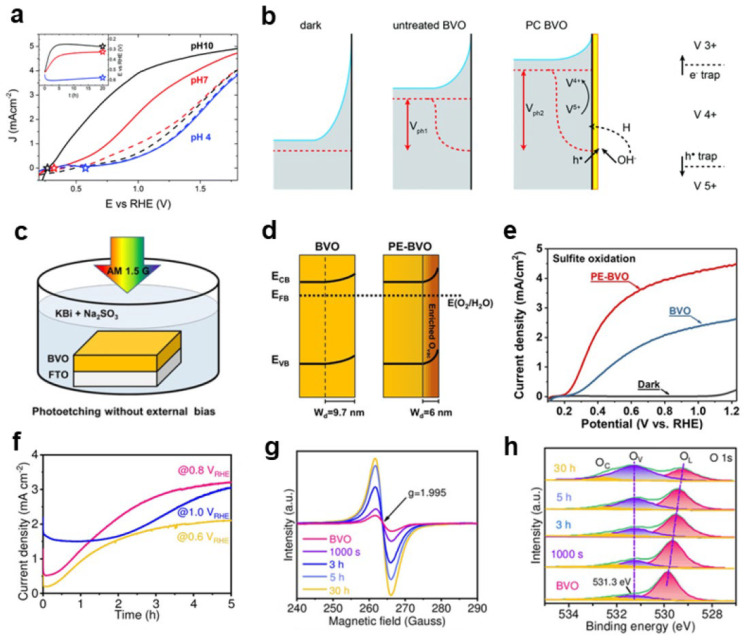
(**a**) J–V curves of BiVO_4_ in PBA buffer at pH 4, 7, and 10; dashed curves–untreated films, solid curves–photocharged films. Inset: open-circuit potential trends collected during the photocharging treatment. Stars in both plots denote the final photocharging open-circuit potential. (**b**) Band diagrams of BiVO_4_ in the dark and under illumination before and after the photocharging treatment, including the simplified photocharging mechanism model. Reproduced with permission [36]. Copyright: 2017, Royal Society of Chemistry. (**c**) Photoetching for the BiVO_4_ photoanode without external bias. (**d**) The band diagrams of BVO and PE–BVO in the dark; (**e**) J–V curves of BVO and PE–BVO measured in 1 M KBi (pH 9.0) buffer solution containing 0.2 M Na_2_SO_3_. Reproduced with permission [37]. Copyright: 2020, Wiley–VCH. (**f**) J–t curves of BVO polarized potentiostatically at 0.6, 0.8, and 1.0 V_RHE_ for 5 h in 1 M KBi (pH 9.5). Characteristics of EL-BVO electrodes polarized potentiostatically at 0.8 V_RHE_ as a function of time (0 s, 1000 s, 3 h, 5 h, and 30 h): (**g**) EPR spectra; (**h**) high-resolution XPS peaks of O1s. Reproduced with permission [39]. Copyright: 2022, Wiley–VCH.

Similar to electrochemical reduction, enrichment of reducing electrons and a subsequent reduction of metal ions can also occur under illumination. Smith et al. proposed a photocharging method to create oxygen vacancies on BiVO_4_ by exposing it to light for 20 h under an open-circuit voltage in a potassium borate electrolyte [35,36]. The photocharging method was dependent on the pH of the electrolyte and only worked under alkaline conditions. The oxygen vacancies generated by photocharging increased the water oxidation current of BiVO_4_ at 1.23 V_RHE_ from 1.0 mA cm^−2^ to 4.3 mA cm^−2^ (Figure 3a,b). However, due to the limited number of holes participating in the reaction, most of the electrons recombined with holes during the photocharging process, and only a small fraction of electrons contributed to the formation of surface oxygen vacancies. Consequently, it takes a longer time to obtain an appropriate amount of surface oxygen vacancies. To accelerate the generation of oxygen vacancies, Gong et al. introduced a hole sacrificial agent, sodium sulfite, to the photocharging solution (Figure 3c). Through a brief 10 min photoetching process, a substantial number of oxygen vacancies were introduced on the surface of BiVO_4_. This method effectively mitigated the detrimental impact of bulk defects. The presence of these surface oxygen vacancies resulted in an increased energy band bending within the BiVO_4_ material. As a consequence, the migration of holes to the surface of the BiVO_4_ photoanode was promoted, facilitating their active participation in the desired reaction. As a result, the photoanode exhibited a higher photocurrent density, indicating improved performance (Figure 3d,e) [37].

Increasing the energy of light can lead to the rapid introduction of oxygen vacancies. Yeo et al. controlled the spatial distribution of oxygen vacancies in WO_3_ photoanodes by a laser-assisted defect control process. Under laser irradiation, the surface of WO_3_ soaked in ethylene glycol solution was quickly reduced and a large number of oxygen vacancies were introduced. Studies have shown that a higher concentration of oxygen vacancies on the surface of WO_3_ than in the bulk is beneficial to the PEC performance of the material [38].

In addition to the methods mentioned above, oxygen vacancies can be introduced during photoelectrochemical testing, enabling in situ regulation of the number of oxygen vacancies on the surface of metal oxides. For example, Wang et al. proposed that potentiostatic photopolarization can significantly improve the photoelectrochemical performance of BiVO_4_. At a potential of 0.8 V_RHE_, the oxygen vacancies and water oxidation activity of BiVO_4_ photoanode gradually increase with the prolongation of photopolarization treatment time (Figure 3f–h). Through the synergistic effect of oxygen vacancies generated by potentiostatic photopolarization and passivated surface states, the recombination of bulk carriers is reduced, water oxidation kinetics are enhanced, and photocorrosion is suppressed [39]. Furthermore, the same group reported that the number of oxygen vacancies in the surface of WO_3_ photoanodes can be tuned by photocharging. It is also proposed that the number of oxygen vacancies tend to reduce during the water oxidation process, resulting in a decrease in its activity, and the activity of the photoanode will be restored after the oxygen vacancies are replenished by photocharging [40].

#### 2.1.2. The Role of Surface Oxygen Vacancies

##### Carrier Separation

Surface oxygen vacancies in metal oxide photoanodes play a crucial role in the charge transport process, particularly in facilitating the participation of holes in surface reactions at the semiconductor/electrolyte interface. In the absence of light, band bending arises as a result of charge accumulation at the electrolyte interface, leading to the formation of a depletion layer. When illuminated, the quasi-Fermi level of electrons or holes can either fall below or rise above the redox potential or the redox couple present in the solution. The degree of band bending in metal oxides is not only affected by the applied potential, but also by the presence of surface substances such as oxygen vacancies.

**Figure 4:**
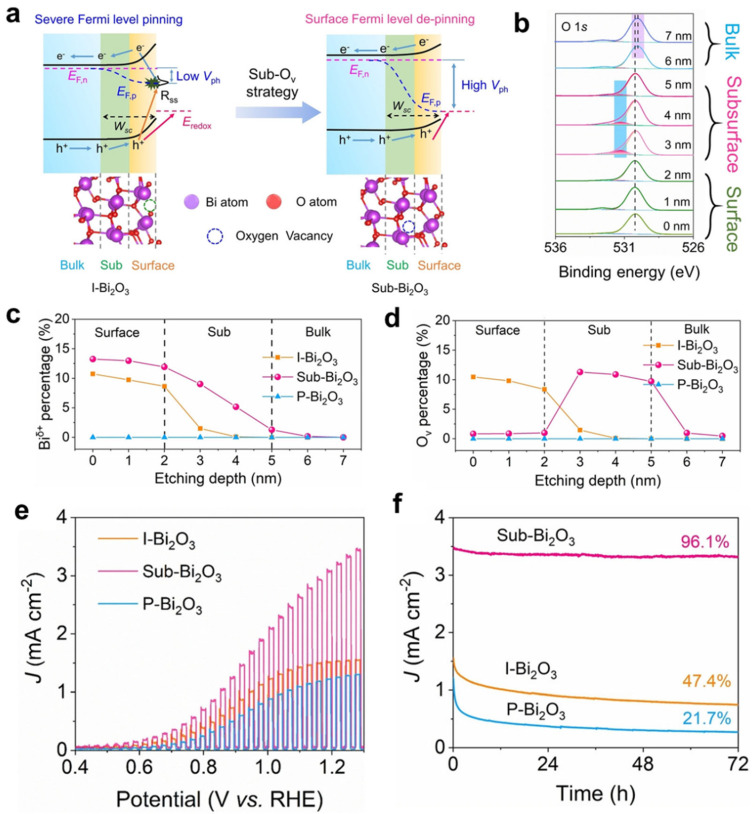
(**a**) Schematic diagram of sub–Ov strategy for Fermi level de-pinning. V_ph_, open–circuit photovoltage; E_F,n_ and E_F,p_, quasi-Fermi level of electrons and holes, respectively; Rss, surface trap state recombination; E_redox_, redox potential of electrolyte; W_sc_, depletion layer width. A Bi_2_O_3_ slab is utilized as the prototype to demonstrate the release of the severe Fermi level pinning effect by introducing O_v_ in the subsurface region. (**b**) O1s orbits for Sub−Bi_2_O_3_ with an etching depth from 0 to 7 nm. Concentration profile of (**c**) low-valence Bi^δ+^ species and (**d**) characteristic O_v_ as a function of etching depth for different samples. (**e**) J–V plots under chopped light and (**f**) steady-state photocurrent for over 72 h on as-prepared samples at 1.23 V_RHE_. Reproduced with permission [43]. Copyright: 2023, Wiley–VCH.

For example, Gong et al. introduced abundant oxygen vacancies on the surface of a BiVO_4_ photoanode, leading to a reduction in the width of the depletion layer from 9.7 nm to 6 nm [37]. This intensified the energy band bending and promoted the migration of holes to the reaction interface. Additionally, gap states introduced by oxygen vacancies can trap holes and slow down charge recombination. In the case of small-sized particle-stacked Fe_2_O_3_ photoanodes, the introduction of surface oxygen vacancies resulted in a depletion layer width of only 1 nm, providing a significant potential gradient to drive the separation of photogenerated electrons and holes [41]. Through a “self-purification” treatment driven by photoelectrochemistry, TiO_2_ exhibited a gradient distribution of oxygen vacancies in the surface layer, within a thickness range of approximately 9.5 nm, achieving a charge transport efficiency of 95% [42]. Theoretical calculations conducted by Wang et al. have revealed the significant impact of subsurface oxygen vacancies (sub–O_v_) on the behavior of oxide semiconductors, particularly in terms of Fermi level de-pinning and enhanced hole migration (Figure 4a) [43]. To verify this, the author developed a series of oxide photoanodes that exclusively possess subsurface holes (Figure 4b–d). In comparison to oxides with surface oxygen vacancies or those lacking oxygen vacancies entirely, the presence of sub–Ov in the oxide photoanodes demonstrated superior activity and stability (Figure 4e, f). The active sub–Ov structure effectively lowers the energy barrier of the rate-determining step during the water oxidation process, thereby enhancing the kinetics of water oxidation.

In addition to affecting the band bending of metal oxides, surface oxygen defects can also change the lifetime of carriers, thereby improving the photocatalytic activity. For example, Sun et al. demonstrated that by gradually increasing the photoelectrochemical polarization time at 0.65 V_RHE_, and the content of oxygen defects gradually increased, leading to an improvement in carrier lifetime [40]. However, during the reaction process, oxygen vacancies tend to be filled and consumed. To address this, the author implemented an intermittent potential to maintain a dynamic balance of oxygen vacancies, ensuring relatively stable activity. However, a drawback of this approach is the rapid filling speed of oxygen vacancies generated by photocharging, resulting in some carriers being underutilized. Gao et al. revealed that the concentration of oxygen vacancies on BiVO_4_ directly influences the carrier lifetime, and the introduction of platinum doping can prevent undesirable charge recombination on defective BiVO_4_ [44]. Similarly, in a study on Fe_2_O_3_ photoanodes, it was also found that the introduction of surface oxygen vacancies leads to longer-lived photogenerated holes, which is more conducive to the oxidation of water [45].

If the holes do not participate in the reaction in time on the surface, recombination can take place. In order to improve the injection efficiency of the photoanode, a hole transport layer is usually required. Among them, β-FeOOH is commonly used as a hole transport layer and an oxygen evolution co-catalyst. When the β-FeOOH nanolayer builds abundant surface oxygen vacancies, it can effectively promote the transport and capture of holes and provide more activity for water oxidation sites [46]. Likewise, in the case of Fe-doped CoO_x_, the introduction of Fe leads to the generation of abundant oxygen vacancies. This, in turn, enhances the transport and capture of holes when FeCoO_x_ is employed as an oxygen evolution co-catalyst. Consequently, the overpotential required for the oxygen evolution reaction is reduced, resulting in a substantial improvement in the photocurrent density and durability of the system [47].

Surface oxygen defects also affect the distribution of their surface states. For example, in the TiO_2_@BiVO_4_ photoanode, the introduction of surface oxygen vacancies leads to an increased accumulation of intermediates with rapid water oxidation kinetics. V_OV_^4+^ acts as a reaction center, facilitating the rapid extraction of holes participating in the reaction. In addition, TiO_2_ passivates the surface recombination center of the BiVO_4_ photoanode, preventing the irreversible surface conversion of electron trapping to VO_2_^+^ to VO^2+^ and enhancing the charge separation [48]. Furthermore, Zhou et al. discovered that the introduction of surface oxygen vacancies in the PbCrO_4_ structure creates both shallow and deep energy levels due to the asymmetry of oxygen atoms [49]. Calcination of PbCrO_4_ under an oxygen-deficient atmosphere generates additional deep energy levels, which induce the formation of more surface trapping states. This phenomenon leads to a higher charge separation driving force, significantly enhancing the charge injection efficiency of the PbCrO_4_ photoanode.

In addition to the benefits of surface oxygen vacancies mentioned above, the adverse effects of surface oxygen vacancies on the photoelectrocatalytic process should also be noted. It has been reported that oxygen vacancies on the surface of WO_3_ act as recombination centers, reducing the charge separation efficiency [21,22]. It was also found in Fe_2_O_3_ [50,51,52,53,54] and CuWO_4_ [55] photoanodes that although oxygen vacancies can promote the surface electrocatalytic process, they can also act as trap states to induce severe interfacial reorganization, resulting in a decrease in charge efficiency.

##### Surface Oxidation Reaction

Surface oxygen defects not only affect interfacial charge separation, as discussed above, but are also critical for surface oxidation reactions. They can influence the adsorption of substrates and reaction intermediates, as well as the kinetics of oxidation steps such as dehydrogenation. A notable example was demonstrated by Wu et al., exhibiting a strong activation of chloride ions across a wide pH range (2–12) on the TiO_2_ photoanode with oxygen vacancies. This activation drives the efficient mineralization of persistent organic pollutants in wastewater, without producing toxic chlorate by-products. The presence of oxygen vacancies on the TiO_2_ surface facilitates the adsorption and activation of chloride ions, leading to the generation of highly reactive chlorine species [34]. Similarly, TiO_2_ with abundant oxygen vacancies can oxidize halide ions to free radicals, which subsequently react with substrates to generate organic halides [19]. Oxygen vacancies can also affect the D-band center of the Fe site on the surface of the Fe_2_O_3_ photoanode, thereby optimizing the adsorption strength of the adsorbed hydroxyl groups and improving the water oxidation performance. A volcanic relationship was found between the activity and the coverage of oxygen vacancies [56].

The presence of surface oxygen vacancies in W-doped BiVO_4_ has been found to enhance the adsorption of OH_ads_, O_ads_, and OOH_ads_ species involved in the water splitting process, thereby promoting the surface catalytic activity [57]. Liu et al. reported the conformal growth of an oxygen-vacancy-rich vanadium oxide on a BiVO_4_ photoanode. This surface modification facilitates the adsorption of water molecules, thereby improving the charge transfer during oxygen evolution reactions [58]. Furthermore, the existence of surface oxygen vacancies in α-Fe_2_O_3_ photoanodes has been shown to improve both their water oxidation activity and stability. These vacancies reduce the width of the depletion layer, while also enhancing the hydrophilicity of α-Fe_2_O_3_. As a result, the water oxidation kinetics on α-Fe_2_O_3_ photoanodes are significantly improved [59].

The presence of surface oxygen vacancies can also affect product selectivity by regulating the reaction pathway. The water oxidation on the oxygen-vacancy-rich WO_3_ photoanode tends to follow a four-electron pathway, leading to suppression of peroxygen species generation. This contributes to a higher Faradaic efficiency in oxygen production and improved stability of oxygen-deficient WO_3_ photoanodes [60]. Park et al. proposed a N_2_-treated surface-oxygen-vacancy-enriched BiVO_4_ photoanode, which tunes the surface wettability of BiVO_4_ toward a kinetically controlled H_2_O_2_ production process. This modification enhances the charge separation efficiency, resulting in a significantly higher Faradaic efficiency (FE) (81.2%) compared to BiVO_4_ photoanodes. At 1.6 V_RHE_, the H_2_O_2_ concentration could accumulate to 4.58 × 10^−4^ M within 2 h, corresponding to a production rate of 11.45 μmol h^−1^. This work highlights the importance of the microenvironment at the surface of photoanodes in mediating competing reactions in aqueous solutions [61]. Theoretical calculations by Pavle et al. demonstrated that the BiVO_4_ (001) surface with subsurface vacancies is most suitable for the oxygen evolution reaction, while the pristine (011) surface is more favorable for the production of H_2_O_2_ [62].

### 2.2. Surface Oxygenated Species

The surface of a photoanode is covered with a large number of adsorbed oxygenated species, some existing before the reaction occurs and some generated during the reaction. Common oxygenated species on metal oxide photoanodes include adsorbed water molecules, adsorbed hydroxyl groups, adsorbed oxygen, peroxygenic species, and oxyanion species. The configuration and composition of oxygen-containing species on the surface of metal oxides are influenced by factors such as the nature of the metal oxide, the preparation method employed, and the reaction conditions. For example, the adsorption properties of water on metal oxides vary due to the inherent differences in the nature of different materials. The surface exposure of the same metal oxide due to different preparation methods also affects the coverage of surface hydroxyl groups. The type and content of oxygen-containing species on the surface of metal oxides can be changed by introducing defects or coating co-catalysts. During the photoelectrocatalytic process, metal oxide photoanodes are usually exposed to electrolytes with water as the solvent. Oxygen-containing species such as water molecules adsorbed on the surface can generate new oxygen-containing species under light or high potential. For example, the adsorbed hydroxyl groups are transformed into hydroxyl radicals with strong oxidizing ability under light irradiation, and the oxoacid anions on the surface of metal oxide photoanodes may be transformed into corresponding anion radicals. These oxygenated species on the surface of metal oxides are crucial for oxidation reactions, especially when the selectivity of oxidation products needs to be controlled.

Oxygenated species on the surface of metal oxide photoanodes affect multiple aspects of the photoelectrochemical process. For example, surface oxygenated species introduce new energy levels at the metal oxide surface, which can act as electron donors or acceptors, thereby influencing the band bending of the material and the overall charge separation process. Furthermore, the adsorbed oxygenated species on the surface of metal oxides have a direct impact on the adsorption and activation processes of reactants at the photoanode surface. Therefore, understanding the classification, generation mechanisms, and roles of these adsorbed oxygenated species is crucial for comprehending their influence on photoelectrocatalytic processes.

In the following section, we delve into the classification of adsorbed oxygenated species on metal oxide surfaces, explore their mechanisms of generation, and elucidate their significance in photoelectrocatalytic reactions.

#### 2.2.1. Water Molecules (H_2_O)

**Figure 5:**
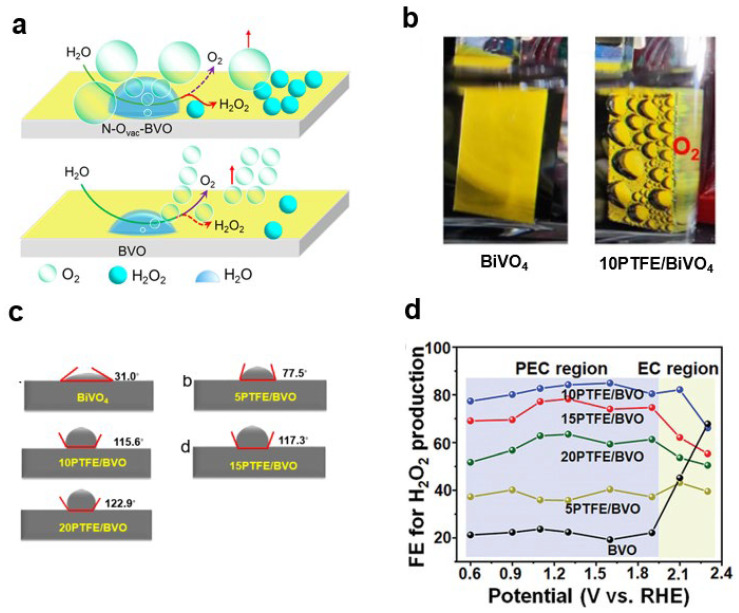
(**a**) Schematic illustrations for tuning the O_2_ gas and H_2_O_2_ liquid product ratios of BVO and N–Ovac–BVO photoanodes. Reproduced with permission [61]. Copyright: 2022, American Chemical Society. (**b**) Digital photos for pure BiVO_4_ and 10PTFE/BVO photoanode at 1.23 V_RHE_ in a 1 M NaHCO_3_ electrolyte. (**c**) Contact angles of liquid H_2_O on BiVO_4_, 5PTFE/BVO, 10PTFE/BVO, 15PTFE/BVO, and 20PTFE/BVO samples. (**d**) The calculated FEs of the PEC H_2_O_2_ evolution of BVO and PTFE/BVO composite photoanodes at various applied biases. Reproduced with permission [65]. Copyright: 2023, Wiley–VCH.

Photoelectrocatalytic water splitting to produce hydrogen and carbon dioxide reduction to produce organic chemicals are mostly carried out in aqueous solutions. In an acidic electrolyte, the adsorption of water molecules on the surface of the photoanode is a prerequisite step for the water oxidation reaction. Therefore, improving the hydrophilicity of photoanodes and enhancing water adsorption are also important strategies to enhance the OER activity of photoanodes. For example, Fe_2_O_3_ has abundant adsorbed hydroxyl groups after solvothermal treatment in methanol, which increases its hydrophilicity and enhances its water oxidation activity [63]. It has also been reported that highly hydrophilic 1-hydroxyethylidene-1,1-diphosphonic acid (HEDP) can be deposited on the surface of the Fe_2_O_3_/Fe_2_TiO_5_ heterostructure. The HEDP-covering layer can be used as a hole storage layer by promoting charge transfer and improving the oxidation kinetics of water [64]. The different adsorption properties of water on the surface of the photoanode will lead to changes in the oxidation reaction path of water molecules. Wan et al. found that the BiVO_4_ photoanode surface with nitrogen doping introducing oxygen vacancies (N–O_Vac_–BiVO_4_) becomes less hydrophilic, which promotes the generation of hydrogen peroxide and inhibits the decomposition of hydrogen peroxide [61]. Therefore, the N–O_Vac_–BiVO_4_ photoanode can realize the selective oxidation of water to hydrogen peroxide (Figure 5a). The crystal facets and oxygen vacancies of BiVO_4_ also affect the thermodynamics of water molecule oxidation [62]. Ou et al. coated a layer of hydrophobic polytetrafluoroethylene (PTFE) on the surface of the BiVO_4_ photoanode. It can be seen from Figure 5b that when BiVO_4_ is coated with a hydrophobic layer material, the surface becomes hydrophobic, leading to the attachment of a significant number of bubbles on the electrode surface during the reaction process. In contrast, the uncoated BiVO_4_ surface facilitates the easy desorption of bubbles. As the amount of hydrophobic material coating increases, the surface of the catalyst changes from hydrophilic to hydrophobic, and the activity of the photoanode shows a downward trend (Figure 5b, c). The FE of the optimized photoanode to produce hydrogen peroxide reaches 81.6%, four times that of the BiVO_4_ photoanode [65] (Figure 5d). Studies have also shown that highly selective water oxidation to hydrogen peroxide can be achieved on a photoanode with a hydrophilic surface in a carbon quantum dot solution, and the FE can reach 93.5% [66].

#### 2.2.2. Hydroxyl Groups (M–OH)

**Figure 6:**
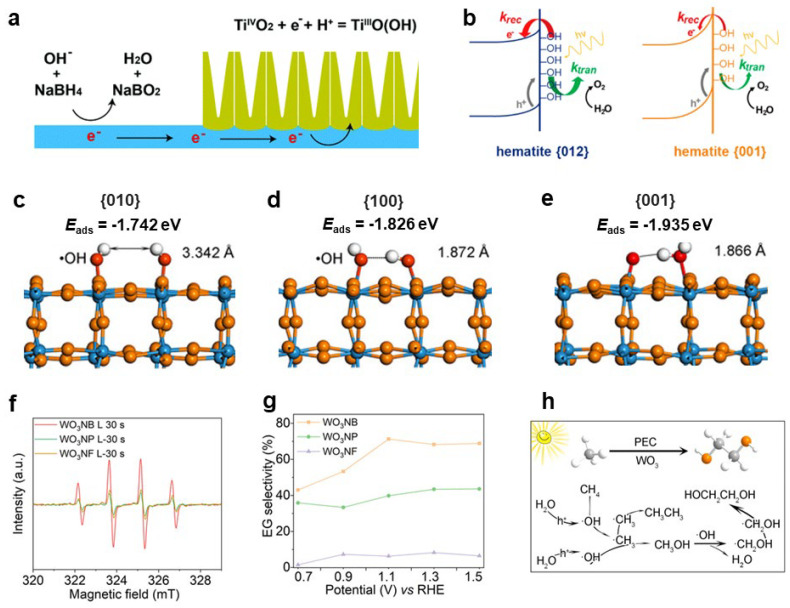
(**a**) Possible mechanisms for the reduction of the TiO_2_ nanotubes via an electrochemical process. Reproduced with permission [68]. Copyright: 2018, Royal Society of Chemistry. (**b**) Schematic illustrating the energetic differences between hematite {012} and hematite {001}. Reproduced with permission [71]. Copyright: 2016, Royal Society of Chemistry. The atomic structures of •OH adsorption on twinning W atoms of (**c**) {010} facet, (**d**) {100} facet, and (**e**) {001} facet. The values at the bottom are their corresponding adsorption energy. The white and red spheres represent the hydrogen and oxygen atoms, respectively. (**f**) EPR detection of OH using DMPO as a spin–trapping agent under 30 s illumination in the presence of WO_3_NB, WO_3_NP, and WO_3_NF. (**g**) Carbon selectivity of EG produced in PEC CH_4_ conversion on WO_3_ photoanodes with different {010} facet ratios at a range of potentials under 100 mW cm^−2^ illumination. (**h**) Schematic illustration of the proposed reaction mechanism for PEC CH_4_ conversion into ethylene glycol. Reproduced with permission [76]. Copyright: 2021, Wiley–VCH.

The hydroxyl groups on the surface of metal oxides are typically produced by the reaction of water or alcohol molecules on the surface. The hydroxyl species act as a bridge between the metal oxide surface and the solution, affecting the reactivity of the surface. They can also act as electron acceptors or recombination centers to affect the charge transfer at the interface. For example, Zhu et al. found that the electrochemical reduction treatment of TiO_2_ nanotubes would induce proton insertion into the TiO_2_ lattice through the reaction of Ti_IV_O_2_ + e^−^+ H^+^ = Ti_III_O(OH) [67]. Photoelectrochemical tests suggest that the surface state (Ti-OH) is an important pathway for photogenerated electron transfer, which can significantly improve the charge separation efficiency of TiO_2_, thus contributing to the enhancement of PEC performance. Similarly, it was also found that fully immersing the TiO_2_ photoanode in the NaBH_4_ solution also generates Ti_III_O(OH) surface states to promote electron transfer, thereby improving the catalytic activity [68](Figure 6a). Notably, the reduction of TiO_2_ by NaBH_4_ becomes challenging when the TiO_2_ photoanode is only partially submerged in the NaBH_4_ solution. Other studies have shown that hydroxyl groups can polarize the surface of TiO_2_, leading to the creation of a substantial electric field within the surface region. This phenomenon results in the elevation of the energy band edge, thus promoting the separation and migration of photogenerated charges, thereby improving the efficiency of water oxidation [69,70]. Similarly, the hydroxyl groups on the surface of the Fe_2_O_3_ photoanode can also act as active sites to mediate the water oxidation reaction [71]. Alternatively, water oxidation can be achieved through hydroxyl radicals, enabling a two-electron oxidation process leading to hydrogen peroxide formation [72]. Nevertheless, the hydroxyl groups on the surface of Fe_2_O_3_ also contribute to charge recombination, thereby diminishing its ability to separate charges effectively [71,73] (Figure 6b). The presence of hydroxyl groups on the surface of ZnO also has an adverse effect on carrier separation [74].

In addition to the influence of charge separation and water oxidation, the conversion of adsorbed hydroxyl groups into hydroxyl radicals for selective oxidation has also been explored. For instance, layered double hydroxides have been found to facilitate the selective adsorption and activation of hydroxyl groups, enabling the selective oxidation of benzyl alcohol to benzaldehyde [75]. By optimizing the adsorption properties of hydroxyl groups and regulating the reactivity of hydroxyl radicals, the oxidative coupling of methane to ethylene glycol was promoted [76]. Ma et al. prepared WO_3_ mainly exposing {010}, {100}, and {001} crystal facets, with nanorods, nanoplates, and nanosheets as the morphology. Experiments and theoretical calculations demonstrated that the {010} crystal facets exhibited the highest hydroxyl adsorption energy and strongest reactivity (Figure 6c–e). Consequently, in the methane oxidation reaction to produce ethylene glycol, where hydroxyl radicals serve as the crucial active oxygen species, WO_3_ nanorods primarily exposing {010} crystal facets displayed the highest activity and selectivity (Figure 6f–h). Additionally, the regulation of the primary and secondary hydroxyl adsorption of substrates can also improve product selectivity [11,77]. Due to the strong oxidizing ability of hydroxyl radicals, the efficient mineralization of substrates is usually achieved by enhancing the generation of hydroxyl radicals in the photoelectrocatalytic degradation of organic pollutants coupled with hydrogen production [78,79].

#### 2.2.3. Adsorbed Oxygen Species (M=O)

**Figure 7:**
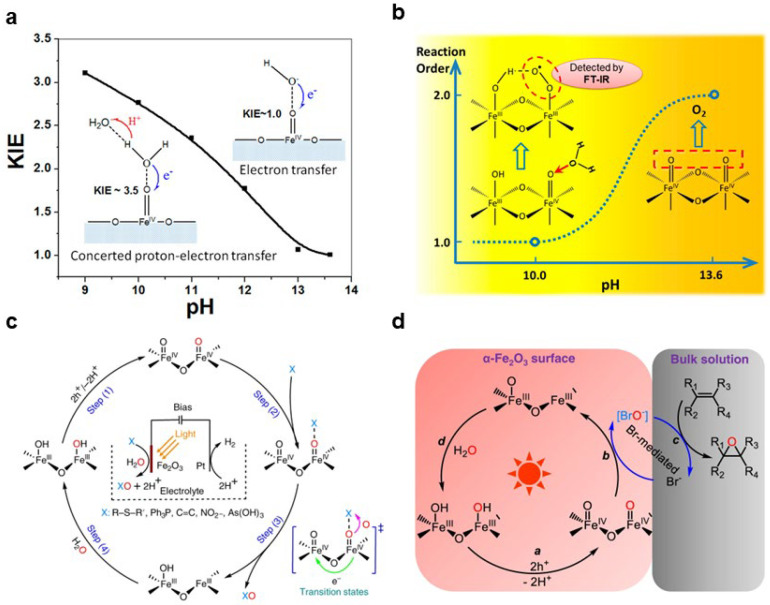
(**a**) The scheme of electron–proton transfer pathways during interfacial hole transfer for oxidation of H_2_O and OH^−^. Reproduced with permission [80]. Copyright: 2016, American Chemical Society. (**b**) Mechanisms for the O–O bond formation on Fe_2_O_3_ surfaces. Reproduced with permission [81]. Copyright: 2018, American Chemical Society. (**c**) The scheme indicates the generation of iron–oxo species (Fe_IV_=O) on α–Fe_2_O_3_ surfaces via photoexcited holes (step (1)). Step (3) describes the transfer of oxygen atoms from the surface Fe_IV_=O to the substrates with a concerted two-hole transfer pathway. Finally, water molecules replenish the consumed oxygen atoms in the catalytic cycle by adsorption and dissociation (step (4)). Reproduced with permission [82]. Copyright: 2021, Nature Publishing Group. (**d**) In the process of Br^−^ mediation epoxidation, the BrO^−^ species are generated via an OAT pathway from Fe_IV_=O species on the surface of α-Fe_2_O_3_, and then transfer the oxygen atom to the alkene in the bulk solution. Reproduced with permission [84]. Copyright: 2023, Nature Publishing Group.

On the surface of metal oxide photoanodes, the generation of adsorbed oxygen on their surfaces typically occurs through the reaction between photogenerated holes and water molecules. Depending on the coordination number and the neighboring atoms, chemisorbed oxygen can be further divided into two subtypes: apical oxygen adsorbed on a metal site and bridging oxygen coordinated to two adjacent metal sites. Adsorbed oxygen is an intermediate in the oxygen evolution reaction process, and its further oxidation process has been widely concerned. The role of adsorbed oxygen on the surface of Fe_2_O_3_ photoanodes has been extensively studied in photoelectrocatalysis. Earlier research revealed that photogenerated holes would generate high-valent Fe_IV_=O species on the Fe_2_O_3_ surface (Figure 7a). The existence of proton acceptors is beneficial to the transfer of electrons, which accelerates the generation of high-valent Fe_IV_=O species. Therefore, maintaining interfacial OH^−^ concentration by buffering bases as proton acceptors can greatly enhance water oxidation activity [80]. Zhang et al. further found that the reaction between water molecules and Fe=O is the mechanism responsible for the formation of O–O bonds under neutral pH electrolyte conditions [81]. In highly alkaline electrolytes, the individual capture of photogenerated holes by two adjacent Fe=O species is the main mechanism for O–O bond formation (Figure 7b). However, under conditions of high photocurrent density, the local pH value of the catalyst surface decreases due to the rapid consumption of hydroxide ions, leading to a transformation in the aforementioned reaction mechanisms.

Additionally, adsorbed oxygen serves as a crucial oxygen atom transfer intermediate for selective oxidation. For example, Zhao et al. found that α-Fe_2_O_3_ is an excellent oxygen atom transfer catalyst [82]. Under mild conditions, it can efficiently oxygenate a variety of substrates using water as the sole source of oxygen atoms, achieving oxidation selectivity and an FE over 90.0% for most substrates. Studies have shown that Fe_IV_=O generated by holes is the key species for the oxygen atom transfer reaction (Figure 7c). Researchers also found that ammonia oxidation occurs on the surface of Fe_2_O_3_ through a non-free-radical-mediated molecular nucleophilic attack mechanism. The selective oxidation of ammonia to NO_x_^−^ and N_2_ can be achieved by adjusting the Fe=O and Fe–N species on the surface of iron oxide [83]. Similarly, Fe_IV_=O of Fe_2_O_3_ is employed for the oxidation of Br^−^ to BrO^−^ through an oxygen atom transfer pathway, and the resulting BrO^−^ transfers its oxygen atoms to alkenes [84] (Figure 7d). This non-radical-mediated and oxygen atom transfer process enables the highly selective epoxidation of various alkenes using water as the oxygen source.

Furthermore, studies also demonstrate that adsorbed oxygen also affects the charge separation performance of the photoanode [85]. For example, Pan et al. found that it is difficult for the adsorbed oxygen to desorb and fill the oxygen vacancies of bismuth vanadate, which reduces the charge separation efficiency. To address this, a protective layer and catalyst composed of NiFe^−^metal^−^organic frameworks (NiFe^−^MOFs) were coated on oxygen-vacancy-rich BiVO_4_ using caffeic acid as a bridging agent. This transfer of oxygen evolution sites from the oxygen vacancy of BiVO_4_ to NiFe-MOFs enhances the activity and stability of the photoanode.

#### 2.2.4. Peroxygen Species (M–O–O)

**Figure 8:**
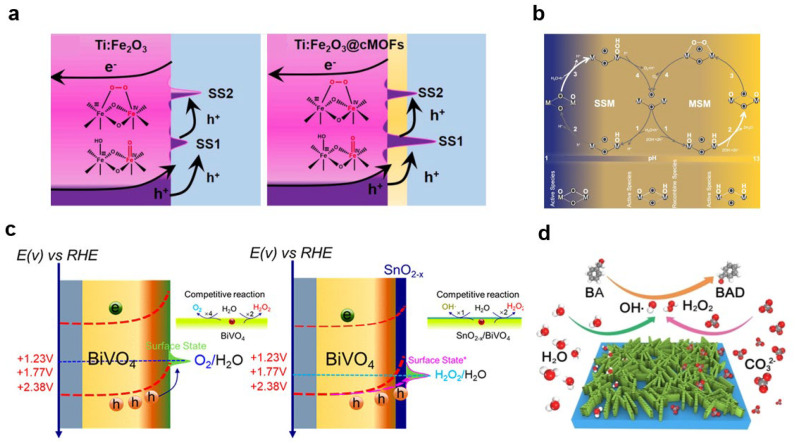
(**a**) Schematic illustration of surface state of Ti: Fe_2_O_3_ and Ti: Fe_2_O_3_@cMOFs. Reproduced with permission [87]. Copyright: 2022, Elsevier. (**b**) Single–site (SSM) and multisite reaction (MSM) mechanisms of OER on metal oxide photoanodes. The white arrows represent the RDS. Reproduced with permission [88]. Copyright: 2023, American Chemical Society. (**c**) SnO_2−x_ overlayer associated with the surface states that are energetically shifted away from the intra-band gap region, which overcomes the O_2_ evolution reaction. Reproduced with permission [72]. Copyright: 2020, American Chemical Society. (**d**) Schematic illustration of selective benzyl alcohol (BA) oxidation process triggered in situ using ROS products [94]. Reproduced with permission. Copyright: 2022, Wiley-VCH.

The oxidation process of water involves the formation of O–O bonds, resulting in the inevitable formation of peroxide species on the surface of metal oxides. These peroxides are undesirable during the oxygen evolution reaction, while in certain cases, hydrogen peroxide is a desired product. However, controlling water oxidation pathways is challenging. Sometimes, these peroxide species not only lead to surface passivation but also introduce recombination centers, reducing the charge separation efficiency and overall photoanode activity. Nonetheless, they can also act as active oxygen species to drive specific selective oxidation reactions. Therefore, it is crucial to investigate the formation mechanism of peroxide species on metal oxide photoanode surfaces. In earlier studies, researchers found that WO_3_ photoanodes produced hydrogen peroxide under acidic conditions and believed that peroxide species would passivate the catalyst surface, resulting in a decrease in the activity of WO_3_. Therefore, when WO_3_ exhibits a high selectivity for oxygen evolution reaction, its stability is also enhanced [86]. The peroxide species on the surface of Fe_2_O_3_ photoanodes are identified as low-energy surface states, which can cause Fermi level pinning and inhibited charge separation performance. We found that the coating of conductive MOF on Fe_2_O_3_ enhanced the high-energy surface state of Fe=O species, thereby enhancing water oxidation activity [87] (Figure 8a). The formation mechanism of peroxide species on the surface of metal oxides depends on the acidity of the electrolyte. In acidic electrolytes, peroxides are usually formed at the single metal site, while in the alkaline solution, adjacent bimetal sites are required [88] (Figure 8b). In order to improve the value of the photoanodic oxidation reaction, more research work has been carried out to improve the selectivity of water oxidation to hydrogen peroxide [89,90,91,92]. For example, passivating the surface of BiVO_4_ by SnO_2-x_ promotes the generation of H_2_O_2_, while inhibiting the competitive OER process [72,93] (Figure 8c). As a reactive oxygen species, hydrogen peroxide can also be used for the selective oxidation of benzyl alcohol [94] (Figure 8d).

#### 2.2.5. Oxyanion Species (RO_X_^n−^)

**Figure 9:**
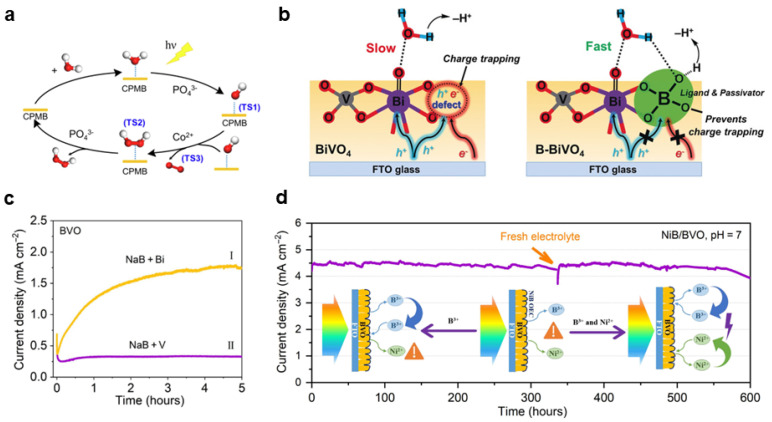
(**a**) Illustration of the possible mechanism of CPMB photoanode for H_2_O_2_ evolution reaction. Reproduced with permission [95]. Copyright: 2022, Elsevier. (**b**) Illustration of the proposed mechanism for water oxidation on the surface of pristine BiVO_4_ and B–BiVO_4_. Reproduced with permission [99]. Copyright: 2019, Wiley-VCH. (**c**) J–t curves of BiVO_4_ (BVO) photo-polarized potentiostatically at 0.8 V_RHE_ for 5 h in NaB + V and NaB + Bi (pH 7). (**d**) A 600 h operation of NiB/BVO applied at 0.8 V_RHE_ in NaB + Ni. The electrolyte was replaced with a fresh electrolyte during the test of 335 h. The inset of (**d**) shows a schematic illustration of B/BVO and NiB/BVO. Reproduced with permission [101]. Copyright: 2023, AAAS.

In addition to the oxygenated intermediates involved in water oxidation in the photoanode, the oxyanion species in the electrolyte also play a key role in the photoanode reactions. Oxyanion species on the surface of metal oxides can promote charge separation and surface reactions through post treatment. For example, phosphate treatment on the surface of BiVO_4_ can improve the Faradaic efficiency of water oxidation to H_2_O_2_, achieving a long-lasting PEC reaction of more than 100 h [95] (Figure 9a). Studies have also shown that phosphate not only promotes the splitting of water molecules into adsorbed hydroxyl groups, but also facilitates the desorption of hydrogen peroxide [95]. Peroxycarbonate, produced by the oxidation of the carbonate, can easily decompose to produce hydrogen peroxide. Thus, it is often used as an electrolyte in the research of hydrogen peroxide produced by PEC [96]. The addition of carbonate can also generate carbonate radicals, accelerating organic matter degradation and generating more active oxygen species for selective oxidation [97]. When Br^−^ ions are introduced into the electrolyte, the BrO^−^ produced by it can also be used for an epoxidation reaction with excellent selectivity [84]. However, the presence of anions is not always advantageous, as anion oxidation may occur instead of water oxidation. For instance, the Faradaic efficiency of tungsten oxide photoanodes for oxygen evolution reaction in acidic sodium sulfate electrolytes is relatively low because most of the holes are utilized for sulfate ion oxidation [98]. In addition, oxyanions also influence charge separation at the semiconductor–electrolyte interface. Immersing a BiVO_4_ photoanode in borate modifies the catalytic local environment and reduces surface charge recombination, resulting in faster water reactions [99] (Figure 9b). The dissolution of semiconductors produces the corresponding oxyanions, and the regulation of the dissolution process is crucial to the stability of the semiconductor–electrolyte reaction interface. For example, bismuth vanadate undergoes continuous leaching of vanadate ions during the reaction process, limiting its long-term stability. By adjusting the composition of the electrolyte, vanadate ions leaching in bismuth vanadate can be suppressed in the vanadate-saturated electrolyte, significantly improving stability [100]. Further studies demonstrated that boron species in the electrolyte could replace vanadate ions after the leaching of vanadate ions in borate solution, resulting in enhanced activity [101] (Figure 9c). By introducing nickel species into the electrolyte, a dynamically stable nickel borate coating layer can be formed on the surface of the bismuth vanadate, resulting in an excellent stability of the prepared photoanode (Figure 9d).

## 3. Characterization Techniques

Characterizing surface oxygen vacancies and oxygenated species involves identifying their composition and determining their role in catalytic processes. Recent advancements in surface characterization techniques have greatly enhanced our understanding of catalyst surfaces. Spectroscopy and electrochemical characterization techniques are particularly valuable for photoanodic surface analysis, enabling in-depth investigations into the properties and behaviors of these crucial surface species. Due to space constraints and the complexity of the operando characterization of surfaces in fluids, this review does not provide a comprehensive discussion of emerging scanning-probe techniques such as STM and AFM. However, readers are encouraged to explore the relevant literature that delves into these techniques in greater detail [102,103,104,105].

### 3.1. Spectroscopy Techniques

#### 3.1.1. X-ray Photoelectron Spectroscopy (XPS)

XPS is a widely used technique for characterizing the surface of materials. It involves analyzing the emitted photoelectron signals generated when X-rays interact with the surface atoms of a material. By examining the composition and valence state of elements on the surface, XPS can provide qualitative information about the surface elements of materials. Each element exhibits characteristic peaks, and changes in the physical and chemical environment of an element can cause shifts in these peaks. The intensity of the peaks is related to the atomic concentration in the sample.

**Figure 10:**
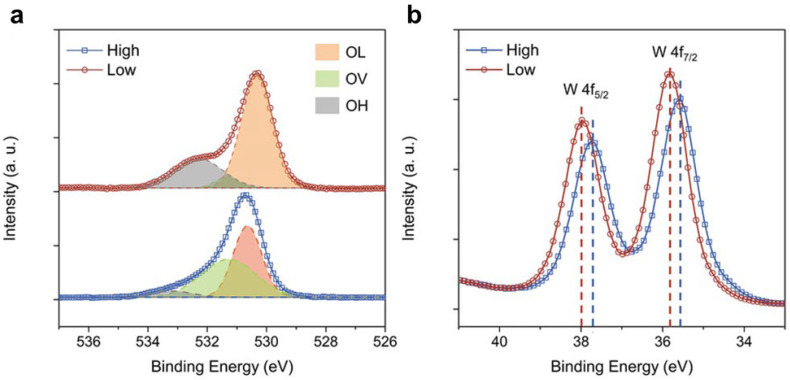
(**a**) O 1s core-level XPS spectra, (**b**) W 4f core–level XPS spectra of the high and low samples. Reproduced with permission [38]. Copyright: 2022, Wiley–VCH.

In the case of metal oxides, when O1s binding energies are observed at higher values compared to the bulk, it is often attributed to oxygen vacancies on the surface. This positive shift in surface atoms indicates a less stable final state compared to bulk excitation. By comparing the peak area ratios corresponding to oxygen vacancies and lattice oxygen in the oxygen characteristic peaks of different materials, the relative number of oxygen vacancies can be obtained [106], for example, in a study by Kong et al. for WO_3_ with a different spatial distribution of oxygen vacancies through laser treatment [38]. By analyzing the photoelectron emission signal of surface oxygen species, the lattice oxygen, oxygen vacancy, and adsorbed hydroxyl group on the surface of the material can be relatively quantified (Figure 10a). Similarly, a shift in the W 4f_5/2_ and W 4f_7/2_ peaks of WO_3_ to lower binding energy indicates the possible presence of more oxygen vacancies (Figure 10b). XPS can also provide information about the distribution of oxygen vacancies on the surface and subsurface of a sample. By comparing XPS spectra of samples with different etching depths, it is possible to obtain information about the presence of gradient oxygen vacancies or other surface features [42,43].

#### 3.1.2. Electron Spin Resonance (ESR)

**Figure 11:**
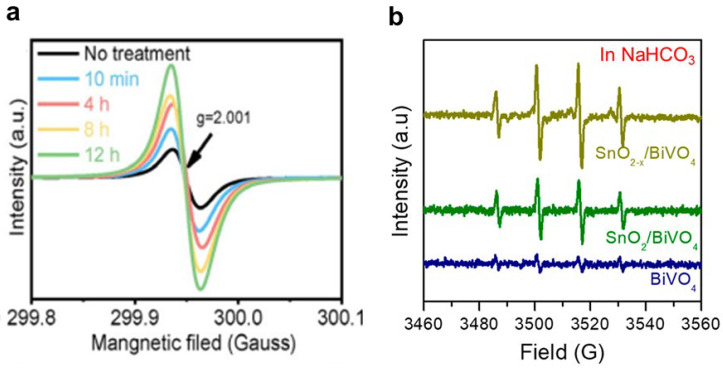
(**a**) EPR spectra of PC–WO_3_ photo-driven at 0.65 V_RHE_ as a function of time in 0.5 M Na_2_SO_4_ under AM 1.5G illumination. (**b**) EPR responses of hydroxyl radical generation by BiVO_4_, SnO_2_, and SnO_2−x_ under visible-light illumination in 1 M NaHCO_3_. Reproduced with permission [72]. Copyright: 2020, American Chemical Society.

ESR spectroscopy is a technique used to directly detect paramagnetic substances that contain unpaired electrons in materials. It is particularly useful for identifying electronic state defects, such as oxygen vacancies, on the surface of a sample. The intensity of the ESR signal provides information about the concentration and type of defects present. Additionally, ESR spectroscopy can be employed to characterize reactive oxygen radicals, including hydroxyl radicals and superoxide radicals. Different types of active oxygen radicals exhibit distinct ESR signal characteristics, enabling the determination of their type and relative concentration through spectrum analysis. For example, Sun et al. studied the surface changes of WO_3_ during the photocharging process [40]. As the photocharging time prolongs, the characteristic peak of oxygen vacancies in the ESR spectrum of WO_3_ photoanode gradually increases, indicating that the number of oxygen vacancies gradually increases (Figure 11a). The characterization of oxygen vacancies can also be further analyzed in combination with two-beam photoacoustic spectroscopy, which enables the investigation of the interaction between oxygen vacancies and the surrounding material matrix [107]. By subjecting the sample to modulated light, the absorbed energy leads to the generation of an acoustic wave that can be detected and analyzed. By comparing the signal from the sample of interest with a reference beam, the specific contribution of oxygen defects to the measured photoacoustic response can be discerned. Zhang et al. found that SnO_2_ with oxygen-defect-coated BiVO_4_ photoanodes exhibits excellent selectivity for the hydrogen peroxide product during water oxidation [72]. The ESR analysis showed that the high selectivity of hydrogen peroxide was attributed to the presence of SnO_2_, which promoted the generation of hydroxyl radicals (Figure 11b). ESR can analyze reactive oxygen species in in situ processes. However, it is important to note that ESR analysis does not provide the absolute quantification of these species.

#### 3.1.3. Ultraviolet–Visible Spectroscopy (UV–_V_is)

**Figure 12:**
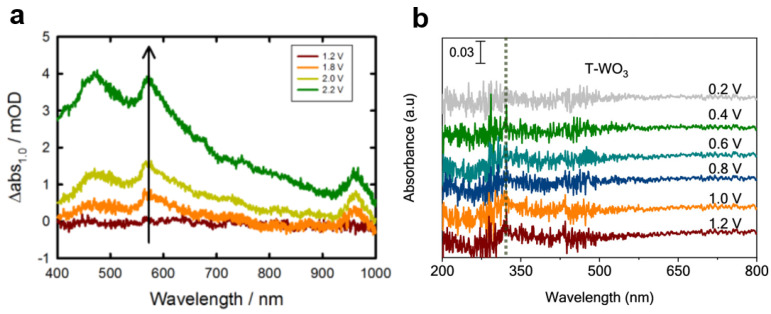
(**a**) Change in absorption spectra measured at different potentials under 0.1 mW cm^−2^ 405 nm illumination. Reproduced with permission [108]. Copyright: 2014, American Chemical Society. (**b**) In situ ultraviolet–visible spectroscopy of T–WO_3_ photoanodes under illumination in 0.1 M NaClO_4_. Reproduced with permission [109]. Copyright: 2023, American Chemical Society.

Ultraviolet–visible spectroscopy is a valuable tool for analyzing oxygen-containing species on the surface of metal oxide photoanodes. By examining the position and shape of absorption peaks in UV–vis spectra, the chemical bonds and functional groups present in the sample can be probed, providing insights into the molecular structure. In order to capture intermediate oxygen species, in situ UV–vis spectroscopy is often performed under reaction conditions. For example, Hamann detected Fe=O surface state species through in situ ultraviolet–visible spectroscopy testing [108]. As the potential exceeds the onset potential of water oxidation at the Fe_2_O_3_ photoanode, the peak area of the ultraviolet absorption peak corresponding to Fe=O surface state species increases gradually (Figure 12a). This surface state was subsequently identified as an intermediate in the water oxidation reaction, highlighting its role in the process. Similarly, Cui employed in situ ultraviolet–visible spectroscopy to detect peroxide species in secondary calcined WO_3_ photoanodes [109] (Figure 12b). The presence of peroxygen species was found to enhance the occurrence of the oxygen generation reaction, resulting in a higher oxygen generation Faradaic efficiency after the secondary calcination of WO_3_. In contrast, tungsten oxide without secondary calcination did not form peroxide species and tended to generate hydroxyl radicals, which were less conducive to oxygen generation. As a result, it exhibited a lower oxygen generation Faradaic efficiency and stability.

#### 3.1.4. Fourier Transform Infrared Spectrometer (FTIR)

**Figure 13:**
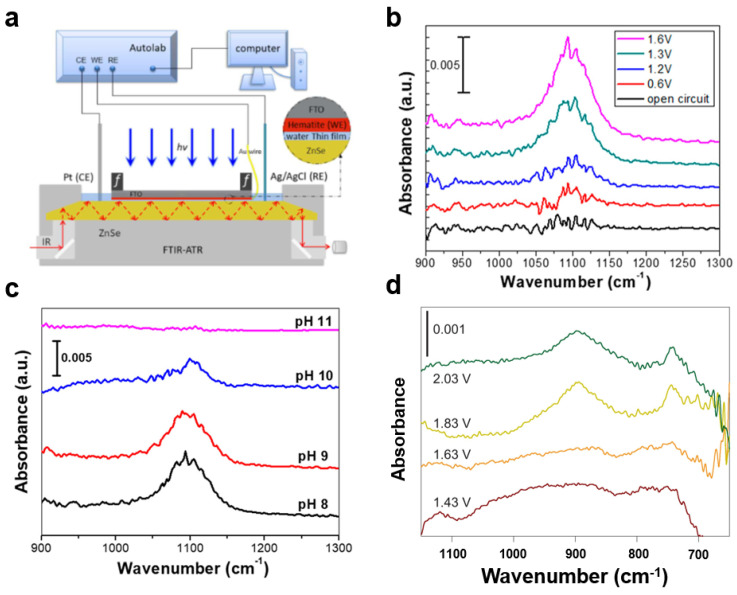
(**a**) Schematic diagram of PEC FTIR–ATR apparatus. (**b**) FTIR spectra on the hematite photoanode under AM 1.5 G illumination in unbuffered pH 8 electrolyte (0.5 M NaClO_4_) with applied potentials from 0.6 V_RHE_ to 1.6 V_RHE_ (**c**) FTIR spectra recorded on the hematite photoanode with applied potential of 1.6 V_RHE_ at different pHs. Reproduced with permission [81]. Copyright: 2018, American Chemical Society. (**d**) Infrared spectra of hematite scanned at constant applied potentials, from 1.43 to 2.03 V_RHE_, in the dark. Reproduced with permission [110]. Copyright: 2016, Nature Publishing Group.

Infrared spectroscopy is a technique that can be used to determine the type and location of oxygen species on the surface of a sample by analyzing the absorption of infrared light of different wavelengths (Figure 13a). Different oxygenated species have different vibration modes and absorb infrared light of different wavelengths. For example, Hamann et al. studied hematite photoanodes under photoelectrochemical water oxidation conditions using in situ infrared spectroscopy. They found that the absorption peak at 898 cm^−1^ belonged to the Fe_IV_=O species, an intermediate of the water oxidation reaction, which suggests that the Fe_IV_=O intermediate was the product of the reaction between the hematite surface and holes [110] (Figure 13d). Similarly, Zhang et al. found superoxides on the surface of Fe_2_O_3_ (Figure 13b). These superoxides result from the bonding of peroxy species to adjacent hydroxyl groups [81]. This confirms the mechanism that water molecules react with Fe=O species to form O–O bonds during water oxidation at near-neutral pH at Fe_2_O_3_ photoanodes. When the pH of the electrolyte increases, the peroxide generation by the one-point water oxidation mechanism gradually decreases, and the reaction gradually changes to a two-site mechanism (Figure 13c). Similarly, researchers have studied the intermediates of TiO_2_, Fe_2_O_3_, and WO_3_ photoanodes during water oxidation using in situ infrared spectroscopy, and detected both low-valent M–OH species and high-valent M=O species on metal oxides [88]. Studies have shown the dual role of the M–OH intermediate. It induces charge recombination in near-neutral electrolytes and acts as an active center for water oxidation under strong alkaline conditions. The study also showed that the water oxidation mechanism shifts from a single-site mechanism to a two-site mechanism as the pH of the electrolyte increases.

#### 3.1.5. Raman Spectroscopy

**Figure 14:**
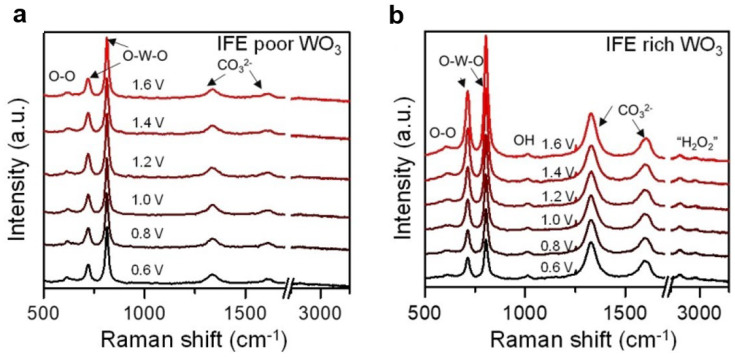
In situ Raman spectra obtained on IFE (**a**) –poor and (**b**) –rich WO_3_ photoanodes; Raman spectra for hematite sample series. Reproduced with permission [94]. Copyright: 2023, Wiley–VCH.

Raman spectroscopy is indeed a valuable technique for characterizing the type and concentration of oxygen species on the surface of materials. By analyzing the scattered light signal generated by incident light interacting with surface molecules, Raman spectroscopy provides insights into the surface properties of the material. The position and intensity of the Raman peaks corresponding to surface oxygen species adsorption can be measured to determine their concentration and type [111]. Furthermore, the presence of oxygen vacancies can cause lattice distortion in materials, which can be qualitatively characterized using Raman spectroscopy [112]. For example, Li et al. utilized in situ Raman spectroscopy to investigate the effect of carbonate ions on the production of reactive oxygen species at a WO_3_ photoanode [94]. The results demonstrated that a WO_3_ photoanode with abundant grain boundaries was more likely to adsorb carbonate ions, leading to an increased generation of hydroxyl radicals and hydrogen peroxide in carbonate electrolytes (Figure 14).

Raman spectroscopy provides valuable insights into the presence of oxygen species, lattice distortion, and the influence of different electrolytes on the production of reactive oxygen species, aiding in the understanding of surface chemistry and the optimization of photoanode performance.

### 3.2. Electrochemical Techniques

#### 3.2.1. Cyclic Voltammetry (CV)

**Figure 15:**
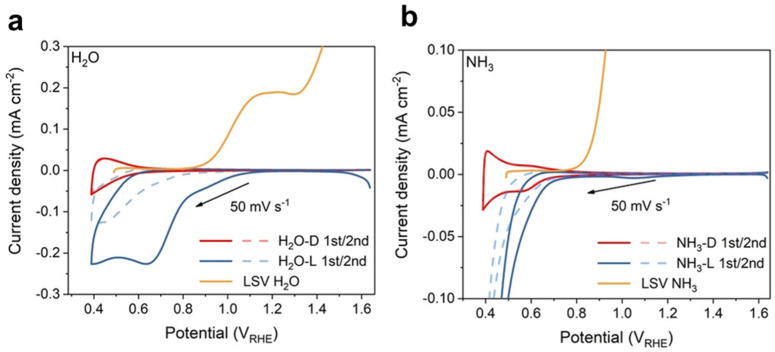
(**a**) Without or (**b**) with 0.1 M NH_3_ after 60 s of polarization at 1.5 V_RHE_ under 470 nm illumination. The solid yellow lines are the corresponding LSV curves. “–L” and “–D” represent that the polarization was conducted under irradiation or in the dark, respectively. The “1st/2nd” represent the first/second cycle of the cathodic CV scans, respectively. Reproduced with permission [83]. Copyright: 2023, Wiley–VCH.

Surface oxygenated species in metal oxide photoanodes exhibit charging and discharging behavior with changes in applied potential, which can be observed through fast cyclic voltammetry scanning. Cyclic voltammetry (CV) scan tests, when combined with photoelectrochemical impedance spectroscopy data, provide valuable information on the presence of surface states. For example, Chen et al. studied the surface state of bismuth vanadate, and they found that during the photocatalytic water oxidation process, a large number of photogenerated electrons participated in the surface reduction process of VO_2_^+^ to VO^2+^, resulting in severe charge recombination [113]. This recombination pathway can be suppressed by the coating of TiO_2_. Further CV analysis revealed that the coating of TiO_2_ can also increase the accumulation of intermediates with fast water oxidation kinetics, corresponding to a surface state of 1.05 V_RHE_. In another study by Wu et al., the ammonia oxidation process of iron oxide photoanodes was investigated [83]. They hypothesized that ammonia would compete with water molecules to react with Fe=O species and confirmed this hypothesis through CV curves. By comparing the CV scanning curves of the iron oxide photoanode after water oxidation and ammonia oxidation, they observed the absence of Fe=O species in the ammonia-oxidized iron oxide photoanode, indicating its involvement as an active oxygen species in the ammonia oxidation process (Figure 15).

The combination of CV scan tests provides valuable insights into the charging and discharging behavior of surface oxygenated species, their participation in redox reactions, and their impact on the photoanode’s performance and reactivity.

#### 3.2.2. Photoelectrochemical Impedance Spectroscopy (PEIS)

**Figure 16:**
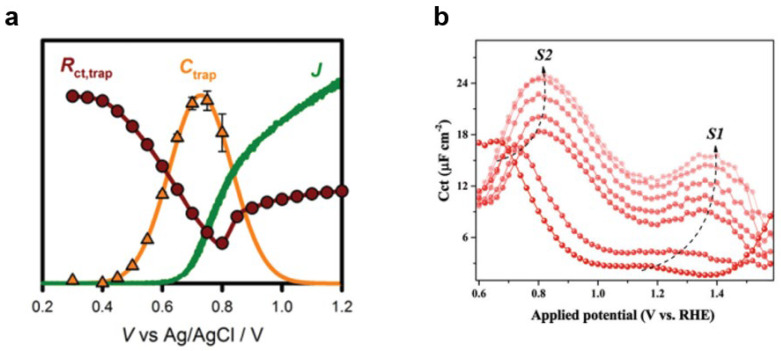
(**a**) J–V curve (green solid line), C_trap_ (orange triangles), and R_ct,trap_ (red circles) values obtained for a 60 nm hematite electrode under 1 sun illumination and pH 6.9. Reproduced with permission [114]. Copyright: 2012, American Chemical Society. (**b**) Evolution of surface state capacitance at stepwise-elevated illumination density (5, 8, 10, 20, 40, 60, and 80 mW cm^−2^, pH 8.0); the growth of S1 and S2 is indicated by arrows. Reproduced with permission [85]. Copyright: 2021, Nature Publishing Group.

The presence of surface oxygen species on metal electrodes can give rise to surface states that influence the capacitive behavior of the electrode. As a result, studying the capacitance behavior as a function of potential provides a valuable approach to investigate and characterize these surface states. By analyzing the changes in capacitance at different potentials, valuable insights can be gained into the nature and impact of surface oxygen species on the electrode’s electrochemical properties. By conducting photoelectrochemical impedance measurements at different potentials and employing equivalent circuit simulations, it is possible to obtain the capacitance versus potential variation curve associated with surface states, determine the potential range governed by the surface states, and analyze the relative characteristics of different surface oxygen species. Klahr et al. performed photoelectrochemical impedance spectroscopy on a Fe_2_O_3_ photoanode and observed that the impedance value initially decreased and then increased as the potential increased [114] (Figure 16a). Through fitting the equivalent circuit, they identified the capacitance corresponding to the surface states. Further investigations revealed the presence of two surface states in the Fe_2_O_3_ photoanode (Figure 16b). Some researchers suggest that the surface state at low potential primarily functions as a recombination center [115], while others argue that both surface states can act as active intermediates in water oxidation [85]. Photoelectrochemical impedance spectroscopy enables the analysis of the influence of surface states on the charge separation performance at the interface.

By combining photoelectrochemical impedance measurements with equivalent circuit modeling, researchers can gain insights into the capacitive behavior of electrode surfaces, understand the impact of surface states on the performance of photoanodes, and analyze the role of surface oxygen species in charge separation processes.

### 3.3. Other Methods

#### 3.3.1. Isotope-Labeling Experiments

**Figure 17:**
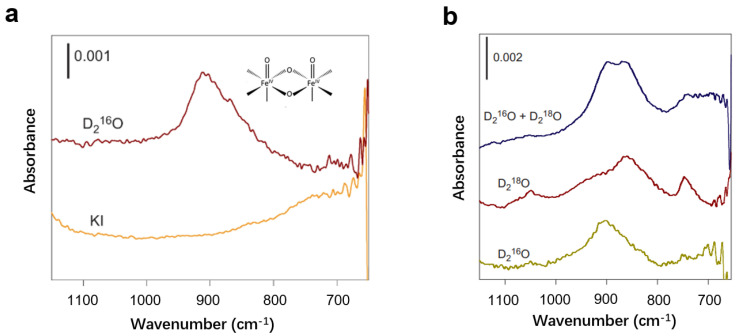
(**a**) The infrared absorption spectra collected at 1.43 V_RHE_ under illumination after turning on the light for a hematite electrode in contact with D_2_O as well as with the addition of KI as a scavenger of valence-band holes. (**b**) The spectra were measured at an applied potential of 1.63 V_RHE_ under illumination in contact with D_2_^16^O, D_2_^18^O, or a 1:1 ratio of D_2_^16^O/D_2_^18^O. Reproduced with permission [110]. Copyright: 2016, Nature Publishing Group.

To determine the specific bonding mode of oxygenated species on the surface, isotope-labeling experiments are commonly employed for analysis. In the case of iron oxide photoanodes, the oxygen-containing species are typically considered to be Fe=O and FeOOH species. To distinguish between these specific species through in situ infrared characterization, researchers utilize isotope-labeling experiments. The authors tested with O^18^ calibrated water and found that the peak of the test was shifted [110]. Further investigations involve testing with aqueous solutions calibrated with both ^18^O and ^16^O. If the surface oxygen species is FeOOH, the in situ infrared spectrum should exhibit three absorption peaks of ^16^O–^16^O, ^16^O–^18^O, and ^18^O–^18^O. However, the measured absorption peak only split into two peaks, and the peaks at 898 and 857 cm^−1^ are assigned to Fe=^16^O and Fe=^18^O, respectively (Figure 17). This analysis allows for the differentiation between Fe=O and FeOOH species. Similarly, the researchers also utilize isotope calibration experiments in the electrolyte at pH 8 to determine the formation of superoxide species from FeOOH and adjacent hydroxyl groups [81]. These experiments provide valuable insights into the specific bonding modes of oxygenated species on the surface of iron oxide photoanodes.

#### 3.3.2. Density Functional Theory (DFT)

**Figure 18:**
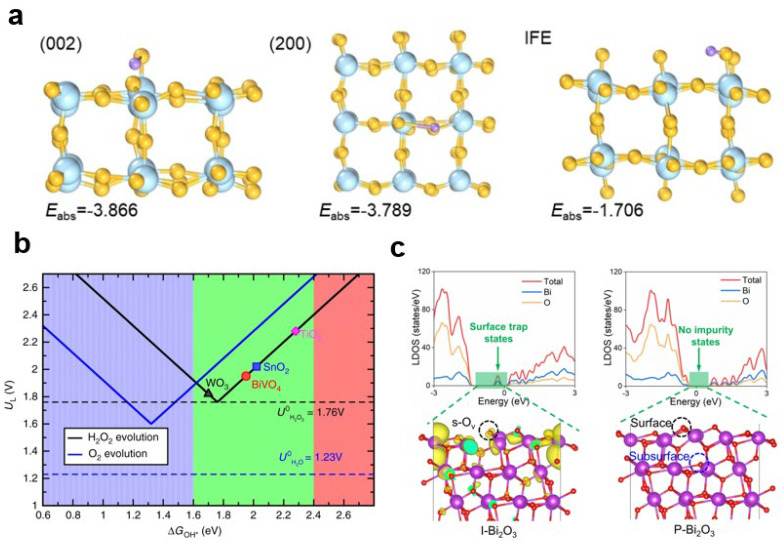
(**a**) The atomic structures of OH adsorption on W atom of (002), (200), and IFE facets. The values at the bottom are their corresponding adsorption energies; purple ball: H atom, yellow ball: O atom, cyan ball: W atom. Reproduced with permission [94]. Copyright: 2023, Wiley–VCH. (**b**) Activity volcano plots based on calculated limiting potentials as a function of calculated adsorption energies of OH* (ΔGOH∗) for the two-electron oxidation of water to hydrogen peroxide evolution (black) and the four-electron oxidation to oxygen evolution (blue). Reproduced with permission [116]. Copyright: 2017, Nature Publishing Group. (**c**) Calculated local density of states and partial charge densities of the mid-gap states for I–Bi_2_O_3_ and P–Bi_2_O_3_. Reproduced with permission [43]. Copyright: 2023, Wiley–VCH.

Density Functional Theory (DFT) is a computational method that can be used to calculate the formation energy of oxygen vacancies on the surface of metal oxides and the adsorption energy of oxygen-containing species. By analyzing these energies, DFT can provide insights into the difficulty of vacancy formation and the effects of oxygen species on charge separation and surface reactions. For example, researchers have used DFT to calculate the adsorption energy of hydroxyl (OH) on different crystal planes and grain boundaries of tungsten oxide, such as the (200) and (002) planes. The results indicated that the adsorption energy of OH at grain boundaries is weaker, suggesting that hydroxyl radicals are more likely to form in these regions [94] (Figure 18a). Calculating the adsorption energy of hydroxyl can also help predict the suitability of different materials for water oxidation to produce hydrogen peroxide (Figure 18b) [116]. By doping bismuth vanadate with Gd, researchers improved the selectivity of water oxidation to hydrogen peroxide. DFT calculations demonstrated that optimizing the hydroxyl adsorption energy promoted the generation of hydrogen peroxide in this system. DFT can also be employed to analyze the effect of oxygen vacancies and surface oxygen species on the charge separation performance of photoanodes by studying the changes in interstitial states. By calculating and analyzing these interstitial states, researchers can gain insights into how oxygen-related defects influence the charge separation capabilities of the photoanode (Figure 18c) [43].

## 4. Summary and Outlook

In summary, the development of efficient and stable photoanodes is crucial for solar energy conversion. These photoanodes should possess a high oxidation reaction activity, excellent charge separation performance and long-term stability, and high selectivity for specific oxidation reactions. However, current research has yet to produce a photoanode that simultaneously achieves high efficiency, selectivity, and stability. Metal oxides, as a promising class of photoanode materials, offer the ability to adjust surface oxygen species, their concentration, and spatial distribution. Modulating these surface properties holds potential for enhancing the performance of photoanodes in terms of activity, selectivity, and stability. With the advancement of surface characterization technology, the understanding of the surface of metal oxides has been deepened. It has been found that the composition and properties of metal oxide surfaces play a key role in the charge separation and reaction of photoanodes. The metal oxide surface not only affects the driving force of hole transfer from the bulk to the surface, and through the interface, but also influences the reaction path and efficiency of the substrate, and the long-term stability of the photoanode. As research in photoelectrochemical (PEC) oxidation extends beyond water oxidation to other oxidation reactions, the development of photoanodes suitable for different oxidation reactions becomes increasingly important. This necessitates a clear understanding of the relationship between surface properties of the photoanode and the specific oxidation reaction. Oxygen vacancies and oxygenated species on the surface of metal oxides are critical surface species that serve as oxidation reaction sites and participate in the transfer of oxygen during the oxidation process of substrates. However, the specific roles of surface oxygen species in oxidation reactions with different substrates are still not fully understood. Therefore, deeper insights into these surface species through advanced surface characterization techniques are necessary to clarify their functions and roles in different oxidation reactions.

(1) Regulation and evolution: The regulation of surface oxygen vacancies and oxygen-containing species often relies on post-processing strategies involving specific atmospheres or solvents, coupled with external stimuli such as heat, electricity, or light. However, the underlying mechanisms governing surface changes during the regulation process remain unclear, and achieving precise control over the spatial distribution and concentration of these surface species remains challenging. Therefore, a deeper understanding of the regulation principles is needed, along with the development of simpler methods to achieve specific control, such as selectively introducing oxygen vacancies and oxygen-containing species on specific exposed crystal faces. Additionally, the stability and differences of surface oxygen vacancies and oxygen species generated by different regulation strategies are rarely investigated. Attention should also be directed toward their evolution during the reaction process, as this may impact their long-term activity. Protection strategies for preserving surface oxygen vacancies and oxygen species should be explored, along with efforts to achieve interface dynamic equilibrium or faster recovery strategies. The dynamic study of surface oxygen vacancies and oxygen species will provide insights into their specific roles in photoelectrocatalytic processes. The evolution study of surface oxygen species will also clarify their specific roles in the photoelectrocatalytic process.
(2) Carrier separation: Surface oxygen species can be either favorable or unfavorable for carrier separation. They can act as charge recombination centers, accelerating charge recombination, or serve as electron donors, leading to Fermi level pinning and inhibiting hole migration, among other undesirable effects. Conversely, they can function as electron or hole capture acceptors, enhancing carrier lifetime and facilitating hole migration. Exploring the fundamental aspects of charge transport and providing a more comprehensive explanation for how surface structure influences electron and vacancy behavior are necessary. For instance, the role of polarons in charge transport and how the introduction of surface oxygen species affects them should be investigated. Furthermore, the influence of surface oxygen species on other material properties should be considered to avoid drawing erroneous conclusions regarding the relationship between catalyst surface structure and charge separation.
(3) Control of product selectivity: The selectivity of oxidation reaction products is affected by various factors, including the adsorption configuration or strength of the substrate on the surface, as well as the energy barriers of different steps in the oxidation process such as dehydrogenation or hydration. Therefore, as the understanding of substrate oxidation reaction mechanisms advances, the role of surface oxygen species in this context should be further explored. Additionally, a clearer understanding of how oxygen species on metal oxide surfaces affect reaction intermediates and products is necessary. Attention should also be given to the influence of different oxygen-containing species on competing oxidation reactions and whether different reaction pathways share common oxygen-containing intermediates. When studying the effect of surface oxygen species on product selectivity, the effect of different metal atoms should be further explored, considering the synergy between single atoms or clusters and surface oxygen species.
(4) For research methods and characterization strategies: To investigate the specific role of surface oxygen vacancies and oxygen-containing species in photocatalytic processes, the development of more comprehensive research methods is essential. These methods should aim to explore their influence on the behavior of molecules and charges. For example, experimental designs can be employed to examine the adsorption performance of surface oxygen species on different substrates or to investigate the adsorption strength of specific functional groups on the catalyst surface. As surface characterization technology advances, finer analyses of surface compositions may become possible. However, the detection of intermediate species for different metal oxides under varying electrolyte or substrate conditions is still lacking. Therefore, it is crucial to employ in situ characterization techniques to detect oxidation reaction intermediates under relevant conditions.

## Data Availability

No new data were created or analyzed in this study. Data sharing is not applicable to this article.
